# Metamorphosis of human lumbar vertebrae induced by VEPTR growth modulation and stress shielding

**DOI:** 10.1007/s11832-015-0677-5

**Published:** 2015-08-11

**Authors:** Carol C. Hasler, Daniel Studer, Philippe Büchler

**Affiliations:** Orthopaedic Department, University Children’s Hospital, PO Box, Spitalstrasse 33, 4031 Basel, Switzerland; Institute for Surgical Technology and Biomechanics, Medical Faculty, University of Bern, Stauffacherstrasse 78, 3014 Bern, Switzerland

**Keywords:** VEPTR, Growth stimulation, Disc narrowing, Vertebral body, Height to depth ratio

## Abstract

**Introduction:**

Distraction-based spinal growth modulation by growing rods or vertical expandable prosthetic titanium ribs (VEPTRs) is the mainstay of instrumented operative strategies to correct early onset spinal deformities. In order to objectify the benefits, it has become common sense to measure the gain in spine height by assessing T1-S1 distance on anteroposterior (AP) radiographs. However, by ignoring growth changes on vertebral levels and by limiting measurement to one plane, valuable data is missed regarding the three-dimensional (3D) effects of growth modulation. This information might be interesting when it comes to final fusion or, even more so, when the protective growing implants are removed and the spine re-exposed to physiologic forces at the end of growth.

**Methods:**

The goal of this retrospective radiographic study was to assess the growth modulating impact of year-long, distraction-based VEPTR treatment on the morphology of single vertebral bodies. We digitally measured lumbar vertebral body height (VBH) and upper endplate depth (VBD) at the time of the index procedure and at follow-up in nine patients with rib-to-ileum constructs (G1) spanning an anatomically normal lumbar spine. Nine patients with congenital thoracic scoliosis and VEPTR rib-to-rib constructs, but uninstrumented lumbar spines, served as controls (G2). All had undergone more than eight half-yearly VEPTR expansions. A Wilcoxon signed-rank test was used for statistical comparison of initial and follow-up VBH, VBD and height/depth (H/D) ratio (significance level 0.05).

**Results:**

The average age was 7.1 years (G1) and 5.2 year (G2, *p* > 0.05) at initial surgery; the average overall follow-up time was 5.5 years (*p* = 1). In both groups, VBH increased significantly without a significant intergroup difference. Group 1 did not show significant growth in depth, whereas VBD increased significantly in the control group. As a consequence, the H/D ratio increased significantly in group 1 whereas it remained unchanged in group 2. The growth rate for height in mm/year was 1.4 (group 1) and 1.1 (group 2, *p* = 0.45), and for depth, it was −0.3 and 1.1 (*p* < 0.05), respectively.

**Conclusions:**

VEPTR growth modulating treatment alters the geometry of vertebral bodies by increasing the H/D ratio. We hypothesize that the implant-related deprivation from axial loads (stress-shielding) impairs anteroposterior growth. The biomechanical consequence of such slender vertebrae when exposed to unprotected loads in case of definitive VEPTR removal at the end of growth is uncertain.

## Introduction

Spinal growth modulation by means of posterior instrumented distraction or anterior tethering is the core component of established and novel methods to control progressive spine deformities in childhood [1–5]. The overall growth stimulating benefits are traditionally objectified by simple measurement of the T1-S1 distance on anteroposterior radiographs [6]. This approach includes the changes of the morphology and severity of the curve during the observational period, the growth of the vertebral bodies and the intervertebral discs. However, T1-S1 values are limited by the projectional nature of a spine radiograph and the ignorance of more detailed regional growth phenomenon. Experimental data support the tremendous remodelling effect of distraction forces on single vertebral bodies exerted by instrumented bridging of multiple spinal segments [7–9]. Distraction forces are even able to promote growth of unilateral congenital bony vertebral bars [10]. Little is known about the effects of distraction-based treatments of early onset spine deformities on the growth and shape of individual human vertebral bodies. A retrospective growing rod case series on twenty patients published in 2012 focused only on the effect of longitudinal growth and a most recent retrospective vertical expandable prosthetic titanium rib (VEPTR) series on 26 children focused on height and width growth of the fifth lumbar and the topmost instrumented thoracic vertebra [5, 11]. In both studies, extra gain in vertebral height growth compared to historical controls was objectified. However, growth in width was diminished. Within the framework of a true non-fusion strategy, a change of the three-dimensional (3D) vertebral morphology might be of biomechanical importance when the spine is re-exposed to full load after removal of the growth-promoting implants at the end of growth. We, therefore, set out to further investigate the effects of vertebral growth modulation looking at individual multiple lumbar levels.

## Materials and methods

**Fig. 1:**
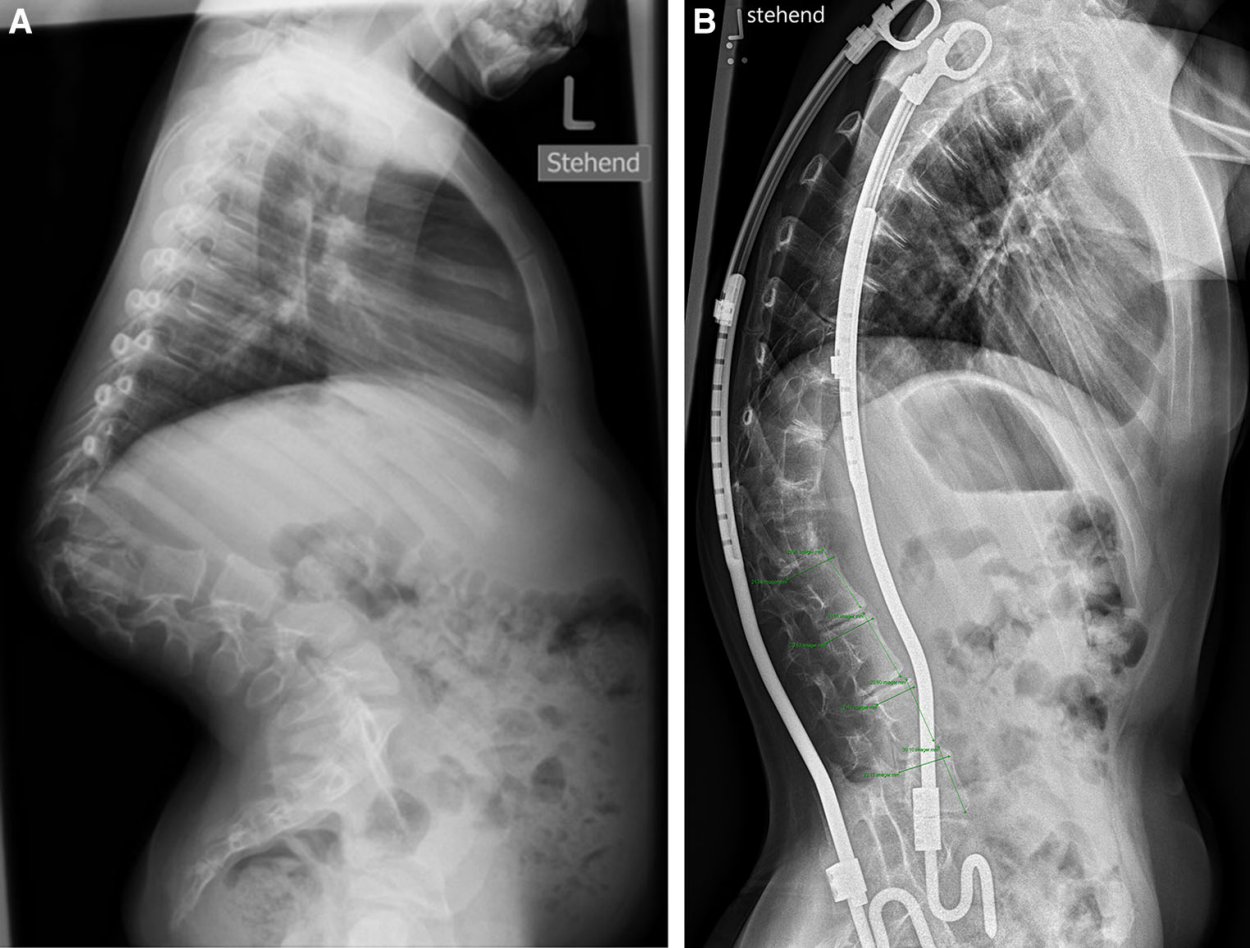
7-year-old girl with a Goldenhar syndrome and a congenital 90° thoracolumbar kyphosis. **a** Prior to the index procedure at the age of 7 years, **b** 6 years after the index procedure and following eleven half-yearly distractions. Tremendous osseous remodelling occurred at the apical level T12/L1, and the vertebral bodies L2 to L5 significantly changed geometry: the height/depth ratio increased by one-third. During the same time the intervertebral discs lost height and the endplates became sclerotic

**Fig. 2:**
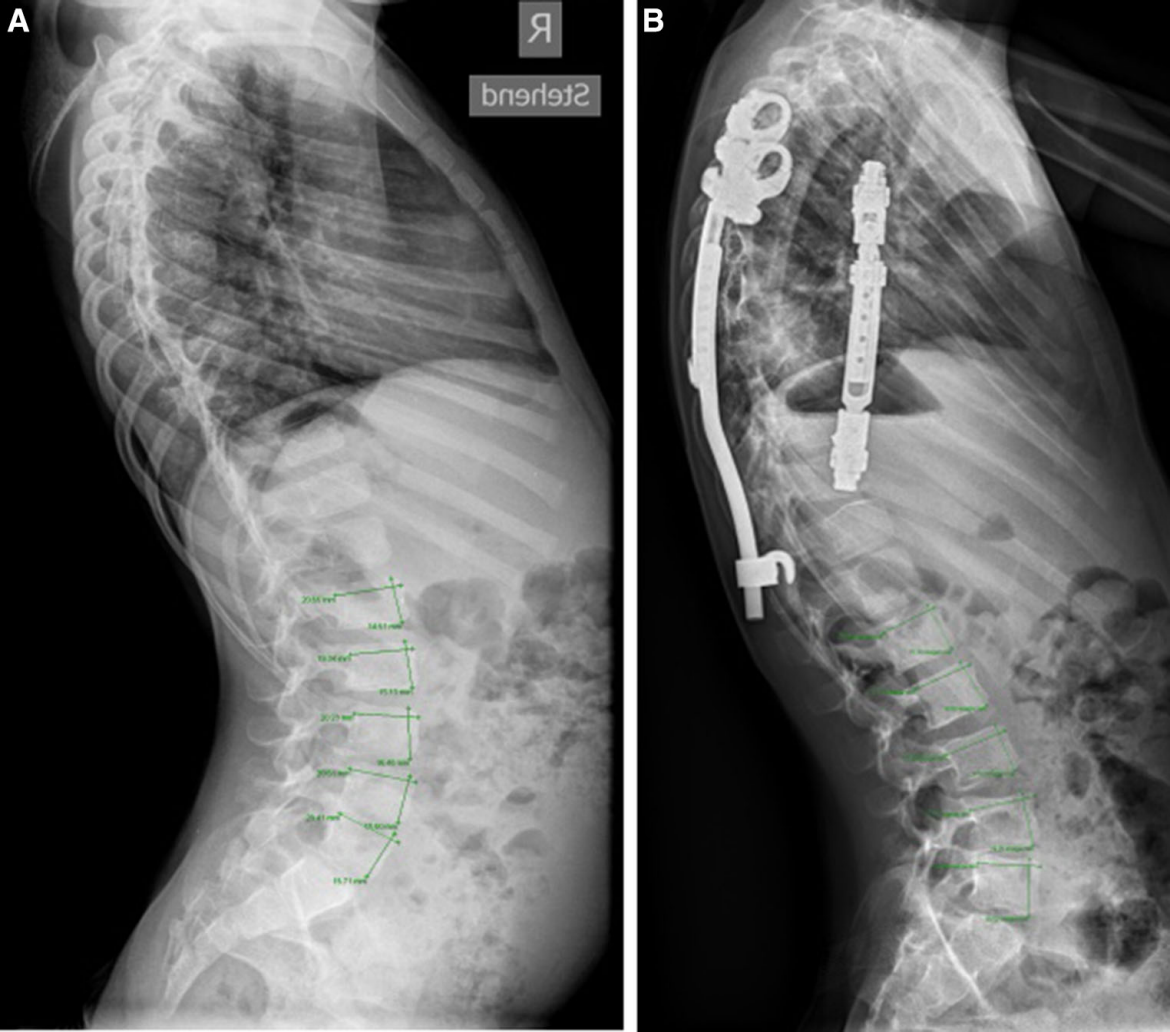
4-year-old boy with congenital thoracic scoliosis treated with classic VEPTR-based expansive thoracostomy. The normally segmented lumbar spine was left alone. The patient, therefore, serves as a comparative control case with naturally developing geometry of the vertebral bodies from prior to the index procedure **(a)** to the follow-up **(b)**. The height/depth ratio and the disc heights remained the same during the 4.5 years of observation

This retrospective radiographic study is based on two groups of nine patients each with early onset spine deformities retrieved from our institution’s database of 61 VEPTR patients. All patients displayed normally segmented and shaped lumbar vertebrae. Group 1 consisted of patients with VEPTR constructs spanning the lumbar spine (Fig. 1). Patients with congenital thoracic scoliosis and rib-to-rib constructs but uninstrumented lumbar spines served as controls (group 2; Fig. 2). They all underwent VEPTR implantation (index procedure) and subsequent half-yearly lengthenings. In order to objectify the growth modulating effect of VEPTR distraction treatment on lumbar vertebral bodies, we measured anterior vertebral body height (VBH) and vertebral body upper endplate depth (VBD) [12] in the sagittal plane of the lumbar spine at the time of the index procedure and at follow-up. According to former morphometric studies, VBH and VBD were directly measured on a PACS client DICOM viewer (CCH) on the last radiographs prior to or after VEPTR implantation and on the last available follow-up radiographs [12, 13]. Those parameters are easily and reliably measurable on a lateral radiograph if the spine is not scoliotic and, therefore, parallel to the film [12]. All images were made at our institution in an upright position following our standard operative procedure for patients with early onset spine deformities. In order to minimize the effect of magnification errors inherent to absolute readings, we added the VBH/VBD ratio to display morphologic changes of the vertebral bodies. Vertebral body endplate width is measured on AP radiographs. Overlapping of anatomical bony structures, excessive lordosis or kyphosis often present in early onset spine deformities have negative impacts on the accuracy of measurement. Therefore, width measurement was not included in this study. The velocity of growth in millimeters per year for height and depth was computed for every patient and vertebra. The measurements were performed on the lumbar spine, the thoracic section being suboptimal due to overlapping of ribs, the smaller vertebral size and the fact that most scoliotic deformities affect the thoracic region. The selection criteria were the followings:

- A minimal follow-up period of more than 4 years was deemed necessary to provide sufficient growth modulation effects
- A non-scoliotic, normally segmented and formed lumbar spine
- At least three lumbar vertebrae not overlapped by implants in group 1
- Image quality allowing for clear delineation of bony landmarks

## Statistical analysis

Since the data were not following a normal distribution, the Wilcoxon signed-rank test has been used for the statistical analysis. The initial dimensions were compared to the vertebral size at follow-up. For each level, group and for the intergroup comparison, the vertebral body height, depth and the ratio between height and depth were analysed. A level of 0.05 has been used to declare statistical significance.

## Results

Nine patients (group 1; six girls, three boys) with miscellaneous underlying spine pathologies (three congenital thoracic scoliosis, three syndromatic spines, one idiopathic early onset scoliosis, one myelodysplasia, one myopathy) were compared to a control group of nine patients (group 2; two girls, seven boys) with congenital thoracic scoliosis. The average age at the time of the index surgery was 7.1 (3–11 years, minimum–maximum) and 5.2 years (1.5–12.8 years), respectively (*p* = 0.1). The average follow-up period was 5.5 years (4.2–6.8 years) and 5.5 years (4.1–7.4 years), respectively (*p* = 1).

**Fig. 3:**
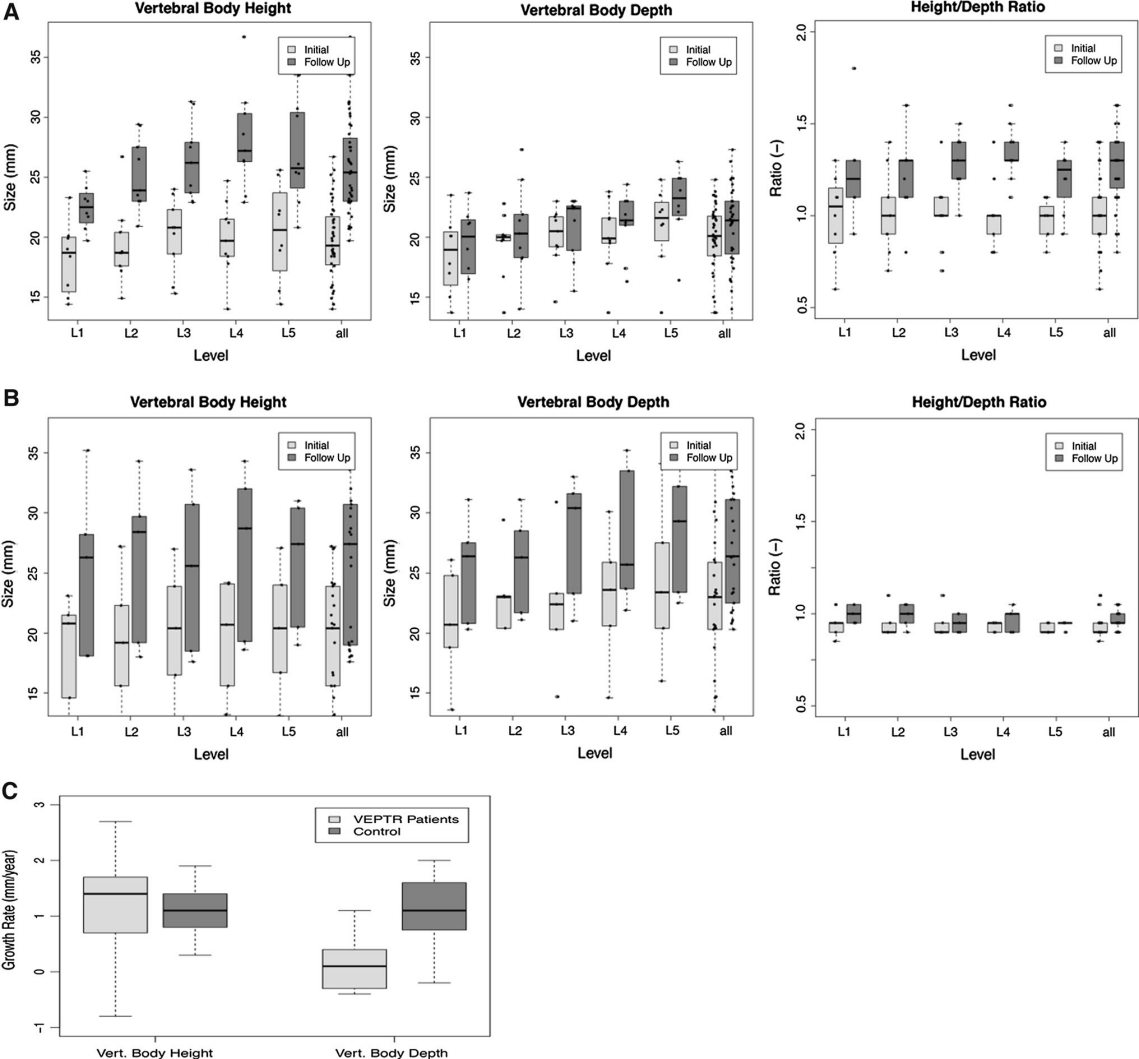
**a** VBH*, VBD** and H/D ratio in group 1, **b** VBH*, VBD** and H/D ratio in group 2 (control group), **c** growth velocities for VBH* and VBD**. *Anterior vertebral body height, **vertebral body upper endplate depth (VBD) [1] in the sagittal plane

Vertebral heights (Fig. 3a) increased significantly in both groups in all lumbar vertebrae. Neither initial nor final heights differed significantly between the groups. The average gain in height was 7.5 (4.2–12.9, group 1) and 6.2 mm (5.4–6.8, group 2) corresponding to a relative increase of 31 % (23–42) and 38 % (33–43), respectively.

The initial vertebral depths did not differ significantly between both groups, but the final depths did (L5 excepted). On all lumbar levels, vertebral depth (Fig. 3b) increased significantly in the control group but not in group 1. The average growth in vertebral depth in the control group was 5.9 mm (5.4–6.7) during the observation period, whereas it almost ceased in group 1, corresponding to a relative increase of 35 % (31–38) and 6 % (6–10), respectively.

H/D ratios (Fig. 3a, b) increased significantly in all lumbar vertebrae in group 1 but not in the control group. The initial ratios did not differ significantly (except L4, *p* = 0.044, and L5, *p* = 0.019 with higher values for group 1) between both groups but the final values did.

Initially, the ratio was 1.0 on average for group 1 (0.6–1.4) and 0.9 (0.7–1.1) for group 2. The relative change up to the time of follow-up was +26 % (21–35) in group 1 and +4 % (−2.5 to 8) in the control group, which resulted in an average value of 1.3 in group 1 (1.3–1.8) whilst it remained 0.9 on an average (0.7–1.1) in the control group.

The growth rate (Fig. 3c) for VBH did not differ significantly between the groups but was significantly smaller for VBD in group 1. VBH growth velocity reached an average of 1.4 mm/year (−0.8 to 8.8) in group 1 and 1.1 mm/year (0.3–1.9) in group 2. VBD growth velocity was almost 0 for group 1 (average of −0.3 mm/year, −4.8 to 3.3) and reached 1.1 mm/year (−0.2 to 2) in group 2.

## Discussion

Growth modulation is the bedrock of non-fusion strategies for the treatment of early onset spine deformities. Anecdotal personal communications of VEPTR and growing rod users on putative changes of 3D morphology of vertebral bodies exposed to year-long instrumented distraction forces oppose to a paucity of clinical studies on that matter. However, those data would help to understand the somehow contradictory biomechanical effects of spinal implants, which keep the spine under distraction over a long period of growth but at the same time immobilize the bridged section. Instrumented spinal distraction entails deprivation from axial loading and presumably from rotatory and bending forces [14]. Morphological and biomechanical changes gain importance in case of implant removal at the end of growth with subsequent re-exposure of the spine to natural forces. Data on 3D physiologic vertebral body growth is astonishingly scarce not to speak of pathologic growth. Longitudinal growth, as provided by two growth plates beneath the vertebral endplates, has been estimated to be between 0.8 mm for a thoracic vertebra and 1.1 mm for a lumbar vertebra per year [15, 16]. Furthermore, there are no data on growth rates of seemingly normal spine sections in patients with congenital anomalies of the spine, e.g., if a normal looking lumbar spine is affected by multiple congenital anomalies of the thoracic spine, mainly if the latter is treated by distraction forces. It is not obvious to what extent and how spine growth occurs in the transverse and sagittal planes of treated and untreated early onset spine deformities.

### Physiologic and growth-modulated change in vertebral body shape

Growing rods and VEPTRs may accelerate longitudinal vertebral growth to double the physiologic levels [5, 11]. In both cited studies, VBH was compared to the physiologic growth data given in the literature, as opposed to our study, with a separate set of similar VEPTR patients serving as controls. Although VBH increased significantly in both our groups to the time of follow-up and the average growth rate of 1.4 mm/year in the segments under distraction clearly surpassed physiologic growth values, the difference to the control group was not statistically significant.

Vertebral body growth in depth almost ceased completely in the lumbar segments spanned by VEPTR implants in contrast to ongoing physiologic growth of about 1 mm/year in uninstrumented controls [17–19]. In accordance to our findings, L5 vertebral body width growth measured on AP radiographs in a series of neuromuscular patients treated by VEPTR also decelerated [5]. Physiologically, H/D ratios remain relatively constant during growth with values slightly below 1 in children under the age of 10 years [17]. In our study, as a consequence of supranormal gains in height and infranormal gains in depth, H/D ratios significantly increased over time in vertebrae exposed to distraction forces, whereas the ratio remained unchanged in the controls. It is well known that growth is biomechanically mediated [7, 9, 20]: distraction forces accelerate and compressive forces decelerate enchondral spinal growth as guided by two physis beneath the vertebral endplates. Growth in depth and width is provided by periosteal appositional growth and may, therefore, continue well into adulthood [21]. In our study, this circumferential growth was negatively affected. It may well be that implant-related stress shielding played the major role. As a result, the overall vertebral shape changed from slightly deeper than high—common in human lumbar vertebrae—to clearly higher than deep—common in quadruped animals—as well documented in comparative anatomical studies [12, 22]. The flattened VBD growth curve during and the VBH/VBD ratio at the end of the observation period in our study patients exactly match the growth dynamics and shape of growing sheep vertebrae and, presumably, also of other quadrupeds [12].

### Observations on vanishing discs

For reasons of inaccuracy related to radiographic measurement of disc heights we did not objectify the height of disc spaces. However, from mere observation and global comparison of the radiographs between the time of the index procedure and the follow-up, it is evident that most disc heights diminished and many endplates became sclerotic over time (Fig. 1) as opposed to the control group where the disc-endplate complexes remained unchanged (Fig. 2). Experimental work in calves and pigs revealed no gross structural changes in harvested narrowed discs after 6 months of anterior spinal flexible tethering [23] and 4 months of distraction with growing rods, respectively [24]. However, this may be different after spanning multiple spinal segments with stiff implants over many years and concomitant degeneration of facet joints. The surgical concept of harnessing growth by growth modulating implants deprives the spine from axial loads and increases axial rotation stiffness in experimental biomechanical investigations in porcine spines [14]. Thereby, it seems to impact upon the biological integrity of the spine and to conflict with the underlying non-fusion strategy. In addition, extraspinal ossifications may also play an important role, as shown in VEPTR patients [25, 26]. This is in line with our and others clinical observations of stiffening spines over time and in concordance with limited correction at the time of conversion into definitive instrumented fusion at the end of growth: this process of autofusion may take place without preceding subperiosteal dissection. Various factors may trigger this process: temporary immobilization of the growing spine by bridging with stiff implants, compressive forces on facet joints as exerted with flexible tethers [6, 27, 28] and even brace treatment may affect flexibility and surgical corrigibility [6].

### Strengths and limitations of this study

There are only few clinical studies focusing on single vertebral growth dynamics during distraction-based instrumented treatment for early onset spine deformities. None of them included vertebral body depth [5, 11]. The number of cases which exhibit a normally segmented, straight lumbar spine spanned by VEPTR rods with a sufficient follow-up period of more than 4 years is limited. As per nature, our cohort displays substantial heterogeneity regarding the underlying spine pathologies but comparison with a VEPTR control group kept the intergroup differences reasonably low.

## Conclusions

Significant 3D morphological changes of individual vertebrae happen with distraction based treatment: deprivation from axial loads (stress-shielding) may lead to extra gain in height at the expense of impaired vertebral growth in depth and width [5]. Hitherto, the biomechanical consequences of this metamorphosis into high and slender quadruped-like vertebral bodies in combination with worrisome disc changes remain unclear, particularly when such altered spines are re-exposed to physiologic forces at the time of metal removal within the framework of a true non-fusion strategy. We are in need of further research on the 3D growth of vertebral bodies in healthy spines, in early onset spine deformities and their morphologic response when exposed to therapeutic distractive or compressive forces.
